# Review of Trackside Monitoring Solutions: From Strain Gages to Optical Fibre Sensors

**DOI:** 10.3390/s150820115

**Published:** 2015-08-14

**Authors:** Georges Kouroussis, Christophe Caucheteur, Damien Kinet, Georgios Alexandrou, Olivier Verlinden, Véronique Moeyaert

**Affiliations:** 1 Department of Theoretical Mechanics, Dynamics and Vibrations, University of Mons—UMONS, Faculty of Engineering, Place du Parc 20, B-7000 Mons, Belgium; E-Mails: geoalexan@hotmail.com (G.A.); olivier.verlinden@umons.ac.be (O.V.); 2 Department of Electromagnetism and Telecommunications, University of Mons—UMONS, Faculty of Engineering, Place du Parc 20, B-7000 Mons, Belgium; E-Mails: christophe.caucheteur@umons.ac.be (C.C.); damien.kinet@umons.ac.be (D.K.); veronique.moeyaert@umons.ac.be (V.M.)

**Keywords:** track safety, strain gage, optical fibre-based sensor, structural health monitoring, strain measurement, track deflection, rail diagnosis, real-time monitoring

## Abstract

A review of recent research on structural monitoring in railway industry is proposed in this paper, with a special focus on stress-based solutions. After a brief analysis of the mechanical behaviour of ballasted railway tracks, an overview of the most common monitoring techniques is presented. A special attention is paid on strain gages and accelerometers for which the accurate mounting position on the track is requisite. These types of solution are then compared to another modern approach based on the use of optical fibres. Besides, an in-depth discussion is made on the evolution of numerical models that investigate the interaction between railway vehicles and tracks. These models are used to validate experimental devices and to predict the best location(s) of the sensors. It is hoped that this review article will stimulate further research activities in this continuously expanding field.

## 1. Introduction

The improvement and modernization of railway networks is a pragmatic solution to congestion issues surrounding larger cities. It represents an interesting modal transfer and, for a long time, multiple studies have been launched with the objective of improving and modernising the rail network. In Europe, various systems have been deployed to adopt a standard in terms of railway traffic (train and embankment adaptation, the European Rail Traffic Management System ERTMS [1], …). Structural health and operation monitoring is one of these continuously developed systems (e.g., [2]). In maintenance, it provides an efficient way to extend the operational life of railway structures. The abnormal state of real structures is predicted using routine measurements and adapted signal processing. In normal operation, it allows detecting the position and kinematics of different trains circulating in a dense network, in order to ensure safe and cost-effective train operations. Current systems, either intrusive or non-intrusive, make use of several sensor technologies. This paper provides a review of recent research investigations about sensors dedicated to the railway traffic and structural monitoring. The focus is made on existing, traditional and proven solutions in addition to the use of fibre-based sensors as a suitable and long-term perspective in the railway sector.

**Figure 1:**
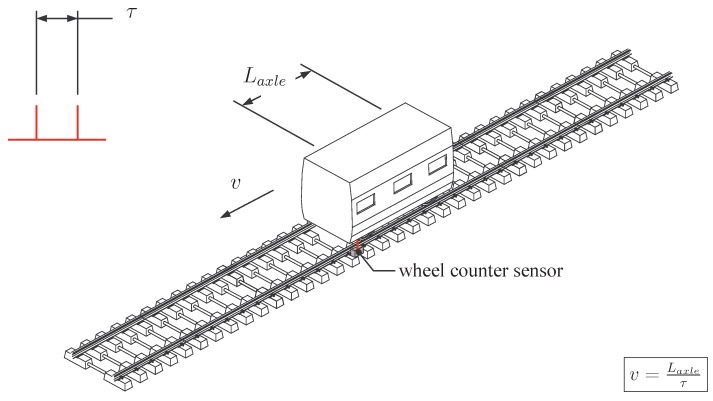
Speed evaluation based on wheel counters.

**Figure 2:**
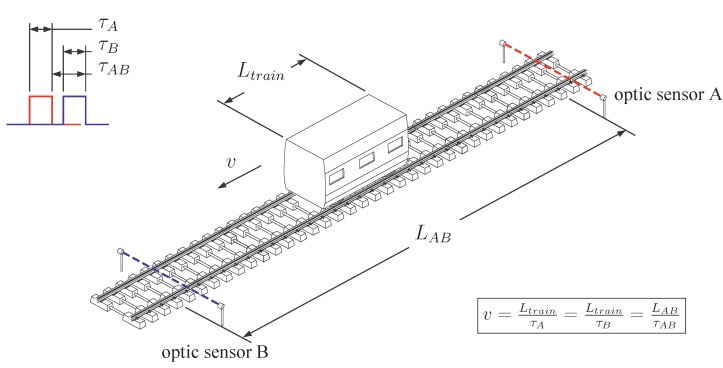
Speed evaluation based on two optic sensors placed along the track.

Historically, conventional monitoring systems in railway infrastructures have been used to assess position and speed of a train. The older method is the track circuit (the first track circuit used in railway signalling has been invented by William Robinson in 1872). It is still used at the present time. The basic principle lies in the connection of the two rails by the rolling stock wheelsets to short out an electrical circuit. This circuit is commonly monitored by electrical equipment (often a relay) to detect the absence of the trains. Another well-known system consist of the wheel counter sensors that are fixed to the rail and detect the passage of train wheels. They can also be used to calculate instantaneous train speed *v* (Figure 1), using the distance between wheels (e.g., the distance $L_{axle}$ inside a bogie). This method therefore needs to specifically know the train type and its geometrical characteristics. The optical photoelectric sensing method is an alternative way to evaluate the train speed. Two devices—a transmitter and a receiver—are placed on both sides of the track, respectively. Using a light beam, the passing of the entire vehicle is detected (Figure 2). The train speed is calculated knowing the train length $L_{train}$ and the time elapsed to cross the sensor. The use of an additional identical system, placed at a sufficient distance $L_{AB}$ along the track, improves the accuracy of the speed calculation. This alternative was used by Ni *et al.* [3] to estimate the speed of vehicles passing on bridge systems in Taiwan and to validate an alternative speed evaluation measurement. Other information is also of interest. Track deflection can be measured in various and independent approaches: monitoring with particle image velocimetry [4] or digital image correlation [5] with the purpose to replace various conventional sensors, geophones mounted on various location of a track to calculate displacements (e.g., on sleepers [4]), multi-depth deflectometers (MDD) and linear variable differential transformers (LVDT) for the deformation of track foundation [6]. Optical detection systems can also be used for evaluating the track deflection. For instance, Pinto *et al.* [7] showed that it is possible to obtain a reasonable accuracy of measuring absolute and relative rail displacements using a position sensitive detector integrated in a continuous monitoring system. A large body of work has been, and continues to be undertaken and this paper will present some interesting and reliable optical fibre-based detection systems.

More recently, a large amount of research in the structural monitoring domain has been undertaken, particularly since the widespread development of high-speed rail lines. Furthermore, the interest of scientific and technical communities continues to grow, with the development of new techniques. Strain gauge sensors were initially considered as the most accurate way to detect the dynamic load and speed of trains, except of some drawbacks: electromagnetic interference, fragility, excessive size, and high dependence on the temperature. New sensors, including fibre technology, present undeniable advantages: high temperature capacity, multiplexing, no sensitivity to the electromagnetic interferences. Moreover, they proved their efficiency in various civil engineering structures [8,9,10,11] and structural monitoring systems other than railway [12]. However, information about installation conditions and interpretation of results is often missing or scarcely described in railway applications.

The present contribution focuses on the state-of-the-art in structural health and operation monitoring systems installed on tracks. A special attention will be paid on new understandings related to stress-based solutions (the term “stress” is commonly used for describing strain condition, explaining why strain sensors are improperly called stress sensors). As a pre-requisite, an essential section describing the mechanisms that contribute to the track dynamics is included. Modelling approaches for numerical simulation, which is useful to calibrate systems in design and development, are presented as well. Moreover, this paper deals with existing conventional and non-conventional measurement systems showing their advantages and possible limitations.

## 2. Static and Dynamic Behaviour of Ballasted Railway Tracks

**Figure 3:**
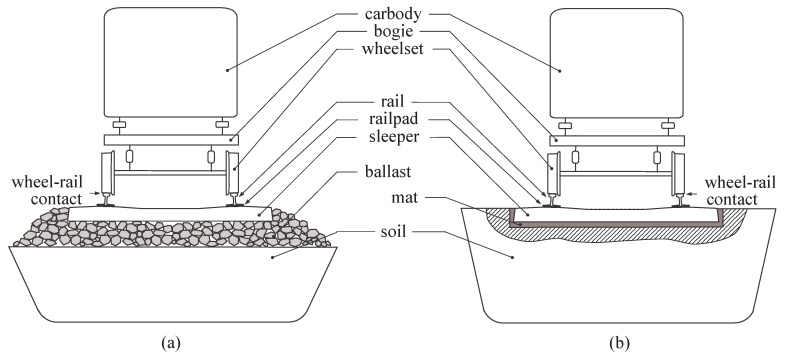
Vehicle/track main components (longitudinal view). (**a**) Ballasted track; (**b**) Slab track.

The vehicle/track system can simply be split into several components as illustrated in Figure 3.

The three main subsystems are the vehicle, the track and the soil, each component playing a different role in the generation of railway dynamics.

1. Related to the vehicle, the suspensions (primary and secondary), carbody, bogie and wheelset masses play an important role in the vehicle vibration modes.
2. Related to the track, various rail profiles and types are available in the railway transport, according to the form, the weight and the track nature. It is noticeable that the standard steel used it typically constant (Young’s modulus: 210GPa and density: $7850\;\mathrm{kg}/m^{3}$). Additionally the geometry varies according to the application and the country. The role of the railpads is to absorb a part of rail vibrations and to allow the wheel to traverse the rail, without damaging the sleeper. The sleeper is another important constitutive element of the track. It has two main roles: to transfer the loads from the rails to the track ballast (or mat) and the ground underneath, meanwhile maintaining the rail gauge. Ballast is used to facilitate the drainage of water and to distribute the load from the railroad ties/sleepers, without distortion by settlement. Ballasted and non-ballasted tracks present similar vertical dynamic behaviour [13]. Therefore, only ballasted railway tracks are considered in the present study.
3. Related to the soil, the dynamic response of foundations subjected to dynamic loadings depends on some key parameters (soil profiles, foundation form and geometry, interaction between adjacent foundations, …) with amplifications and/or attenuations as a function of the excitation frequency [14]. influencing the response.

In an attempt to categorize the vibration signatures of the vehicle, the track and the soil in three frequency bands, Alias [15] proposed that they could be divided into frequency ranges with fuzzy limits:

- Vehicle dynamics intervene in the low-frequency range (until 15Hz) and are efficiently transmitted to the ground if significant defects in the wheel/rail contact excite the vehicle natural modes.
- Mid range frequencies (from 15Hz to 150Hz) are due to the track flexibility with possible amplification due to the soil resonance.
- High-frequencies (over 150Hz) constitute rolling noise due to the wheel/rail sliding and rarely intervene in the ground vibrations because the soil strongly absorbs the vibrations (material and geometrical damping).

To analyse vibration levels, it is first important to be able to understand, and predict vibration levels. Therefore as a first approximation, the passage of a train consists of a number of similar events, each with individual delay times. If a single wheelset moving at speed *v* is considered, its effect on the track can be represented by

(1) $$\begin{array}{c} f\left(t\right)=P_{wheel}\;\delta \left(t-t_{k}\right) \end{array}$$

where $P_{wheel}$ is the nominal loading of a wheel (considered as constant and often expressed in kN), δ(t) the Dirac function and $t_{k}=\frac{x_{k}}{v}$ with $x_{k}$ the position of the impulse load. Equation (1) is defined for both time *t* and distance along the track *x* since x=vt. Its Fourier transform is given by

(2) $$F\left(f\right)=\int_{-\infty }^{+\infty }P_{wheel}\;\delta \left(t-t_{k}\right)e^{-j2\pi ft}dt=P_{wheel}\;e^{-j2\pi ft_{k}}\;$$

**Figure 4:**
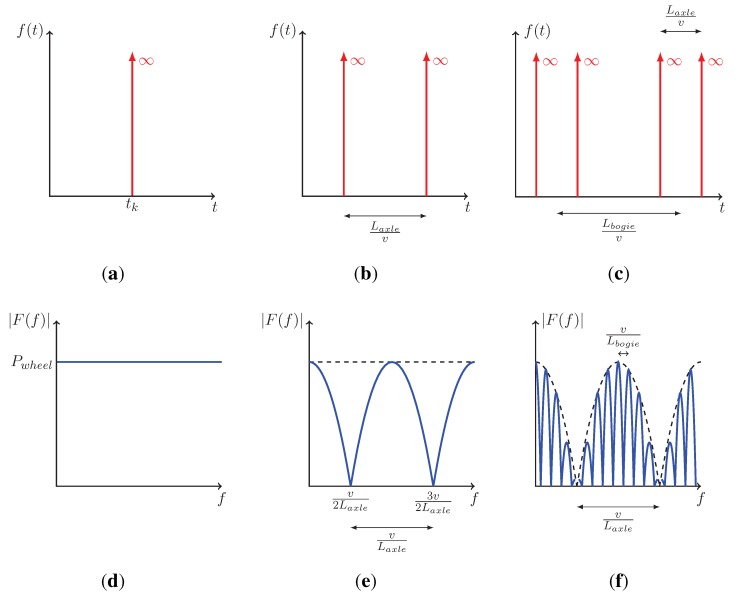
Carbody effect with Dirac functions. (**a**) Single Dirac function—one wheel effect (time history); (**b**) Double Dirac function—two wheels effect (time history); (**c**) Quadruple Dirac function—four wheel effects (time history); (**d**) Single Dirac function—one wheel effect (frequency content); (**e**) Double Dirac function—two wheels effect (frequency content); (**f**) Quadruple Dirac function—four wheel effects (frequency content).

Figure 4a,d display both representations of the Dirac function, showing the expected constant magnitude as a function of the frequency *f*. If two impulse loads are delayed by a distance $L_{axle}$ (Figure 4), the time effect and the corresponding frequency spectrum are given by

(3) $$\begin{array}{ccc} f(t) & = & P_{wheel}\;\left[\delta \left(t-t_{k}\right)+\delta \left(t-t_{k}-L_{axle}/v\right)\right] \end{array}$$

(4) $$\begin{array}{ccc} F(f) & = & P_{wheel}\;e^{-j2\pi ft_{k}}\left(1+e^{-j2\pi f(L_{axle}/v)}\right)\; \end{array}$$

**Figure 5:**
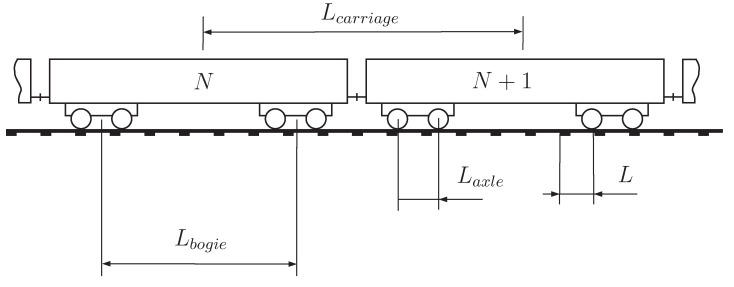
Rudimentary geometrical parameters of the train and the track ([17]).

Compared to Equation (2), the result given by Equation (4) and plotted in Figure 4 shows an amplitude modulation with a beating of $f_{a}=\frac{v}{L_{axle}}$ and zero amplitude at frequencies $\frac{2k+1}{2}f_{a}$ (k∈N). This situation represents the effect of a single bogie (Figure 5) moving at speed *v*. For a complete carbody, the effect is defined as

(5) $$\begin{array}{ccc} f(t) & = & P_{wheel}\;[\delta \left(t-t_{k}\right)+\delta \left(t-t_{k}-L_{axle}/v\right)\\ & + & \delta \left(t-t_{k}-L_{bogie}/v\right)+\delta \left(t-t_{k}-\left(L_{axle}+L_{bogie}\right)/v\right)] \end{array}$$

with the corresponding Fourier transform

(6) $$\begin{array}{ccc} F(f) & = & P_{wheel}\;e^{-j2\pi ft_{k}}\left(1+e^{-j2\pi f(L_{axle}/v)}\right)\left(1+e^{-j2\pi f(L_{bogie}/v)}\right) \end{array}$$

by introducing the bogie distance $L_{bogie}$. Figure 4 illustrates the effect of modulation induced by a second bogie implying that each lobe of width $f_{b}=\frac{v}{L_{bogie}}$ follows the envelope initially defined by Equation (4). By taking into account the number $n_{c}$ of carriages, Equation (6) becomes

(7) $$\begin{array}{c} F\left(f\right)=P_{wheel}\;e^{-j2\pi ft_{k}}\left(1+e^{-j2\pi f(L_{axle}/v)}\right)\left(1+e^{-j2\pi f(L_{bogie}/v)}\right)\left(1+\sum_{i=1}^{n_{c}}e^{-j2\pi if(L_{carriage}/v)}\right) \end{array}$$

and introduces the carriage excitation frequency $f_{c}=\frac{L_{carriage}}{v}$ corresponding to dominant frequencies where the maximum amplitudes follow the envelope. This evaluation of train loading provides a comprehensive interpretation of track response frequency content, including the amplitude modulation which is complementary to the work of Ju *et al.* [16].

The aforementioned analysis is based on a perfectly rigid contact between the vehicle and the track. To take into account the track dynamic behaviour, the track receptance is classically calculated (frequency response function between the track deflection and a stationary input force). This mathematical representation describes the propagation of energy through a system for a set range of frequencies. It is used because it provides a useful tool for investigating the flexibility of a track, as a function of natural frequencies and damping attenuation of the track. In other words, the track/foundation flexibility coupled to the vehicle speed plays an important role on the frequency content of the wheel loads: the wheel/rail force is not strictly similar to a Dirac pulse and has a finite duration dependent on the track flexibility and inversely proportional to the vehicle speed [18]. The modulation effect is therefore observable in a limited frequency range where the magnitude is more or less constant.

Knowledge of amplitude modulations and key excitation frequencies makes it more straightforward to understand the track dynamics. Other phenomena can be added to the dominant frequency spectrum [18]:

- The vehicle dynamics can amplify the spectrum at low frequencies where the vehicle modes interact with the track when rolling on non-perfect surfaces (distributed unevenness or local defect like turnouts and wheel flats). This may amplify the excitation passages frequencies.
- The vertical track dynamics is mainly affected by three resonances: a first resonance where the rail and sleepers vibrate vertically in phase (typically at a frequency around 50–300Hz), a second resonance where rail and sleepers vibrate out of phase (at a medium frequency in the range 200–600Hz) and a third mode, called the pinned-pinned resonance, where the rail vibrates with a wavelength equal to two sleeper bays (close to 800–1000Hz).
- The ground provides two kinds of attenuation: geometric damping and material soil damping. This refers to the exponential decrease of vibration magnitude with distance and to an attenuation at high-frequencies, respectively. Moreover, if the ground is considered as a superposition of layers with different dynamic properties, a resonance can appear if the difference in rigidity between the two top layers is significant and the excitation acts in the vertical direction [19]. The corresponding frequency can be approximated by [20]
  (8) $$\begin{array}{c} f_{soil}=\frac{c_{p}}{4\;h} \end{array}$$
  with $c_{P}$ the soil longitudinal wave velocity and *h* the depth of the soil first layer. Practically, this resonance occurs in a frequency range between 20 and 60Hz depending on the site configuration. This mode is highly damped, meaning that the resonance area covers a large frequency band.

**Figure 6:**
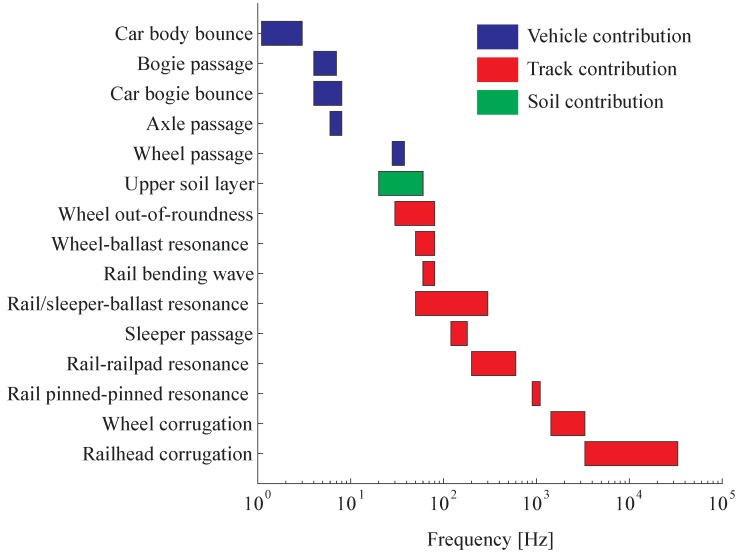
Main contribution of dynamic vehicle/track and soil interactions (with permission from [21]).

All these phenomena are gathered in Figure 6 in relation to the frequency range of interest, according to [21].

Although a sequence of travelling axle loads is a simplified representation of the effect of a moving vehicle on a track, it is commonly used because it provides general information on the vehicle/track dynamics. When the vehicle speed is lower than critical velocities, and as long as the dynamic vehicle/track interaction is low, the effect of moving loads is very similar to the static contribution. This is why the effect is called “quasi-static”. On the contrary, dynamic effects correspond to the following two situations:

- An increased track deflection can appear when the vehicle speed is close to critical track/soil velocities. The physical interpretation of these effects is similar to resonances [13,22].
- In practice, rail and wheel surface imperfections (distributed rail and wheel unevenness, roughness of the wheel and rail surface, out-of-roundness of the wheel, wheel flat, wheel seized ball bearing and other singular track defect like switches, crossings and rail joints) shape up the wheel/rail forces, introducing a fluctuation around the static value.

## 3. Estimation of Rail Stress and Monitoring Assessment Using Numerical Models

**Figure 7:**
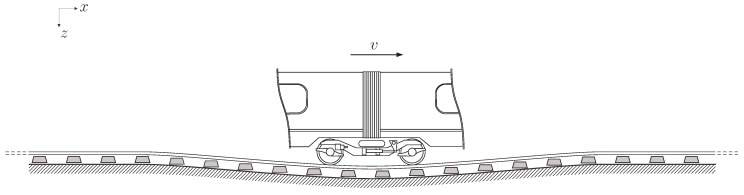
Track deflection generated by the passing of a train.

Simulating a train passage requires modelling track dynamic stress and vibration propagation through a track structure (Figure 7). The frame orientation is recommended as follows: *x* for horizontal parallel to the track, *y* for horizontal perpendicular to the track and *z* for vertical downward. When attempting to model track vibration, the complex wavefields generated by the three-dimensional track geometries (e.g., sleepers and ballast) can hardly be modelled using direct analytical expressions. To overcome these challenges, analytical and numerical approaches make some assumptions regarding the track geometry and components.

**Figure 8:**
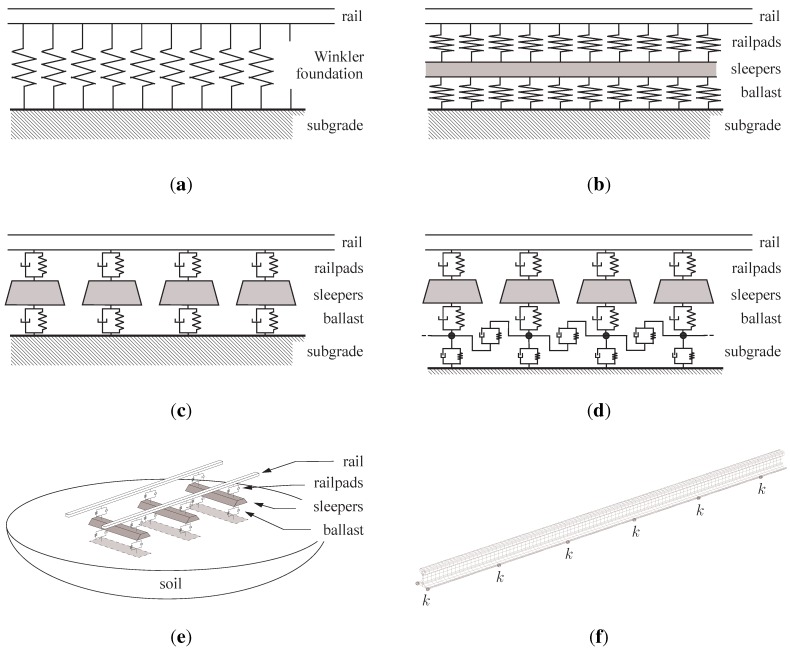
Track modelling: a review. (**a**) 2D single layer track model (continuously supported); (**b**) 2D two layer track model (continuously supported); (**c**) 2D two layer track model (discretely supported); (**d**) 2D three layer track model (discretely supported); (**e**) 3D three layer track/soil model (discretely supported); (**f**) 3D model for the rail (discretely supported).

Figure 8 presents various track models used in analytical and numerical solutions to compute track dynamics. As the vertical loading dominates the dynamic track response, the simplified track structure is most often defined as a bi-dimensional model in the vertical plane along the track (a similar classification can be done in the horizontal plane for lateral loading). Two categories of tracks are proposed, depending on whether the rail is assumed to be continuously or discretely supported. This distinction is imposed by the discrete nature of sleepers along the track direction. Continuously supported models are intended to simulate the entire track and neglect the effect of sleepers. On the contrary, sleeper effects can be modelled using a discontinuous support, which increases the accuracy at higher frequencies. In both cases, the rail is considered as a flexible beam which is either finite (the problem is solved in the time domain) or infinite (in the frequency/wavenumber domain). One of the most straightforward approaches to rail modelling is to use an Euler beam (this modelling approach allows calculating the load-carrying and the small deflection characteristics of a beam). However, Grassie *et al.* [22] concluded that this model is deficient in several aspects in the high frequency range (>100Hz). This was confirmed by [23] by comparing several numerical models. An alternative approach is the Timoshenko beam, a more general theory including shear deflection and rotational inertia of the rail [24] (Euler beam theory is a special case of Timoshenko beam theory). Several layers are used in the model to distinguish the masses of each component (sleeper, rail, ballast, foundation). It is well admitted by the scientific community that the dynamic behaviour of the elastic elements (railpads and ballast) is complex but they can be generally assumed to be massless and are introduced as elastic components, with linear stiffness and damping properties in many applications. Alternatively, the ballast may be included by introducing an additional layer by volume continuity models where the ballast is considered as elastic linear, using discrete element modelling approaches [25] or with additional mass, spring and damper elements [26].

**Figure 9:**
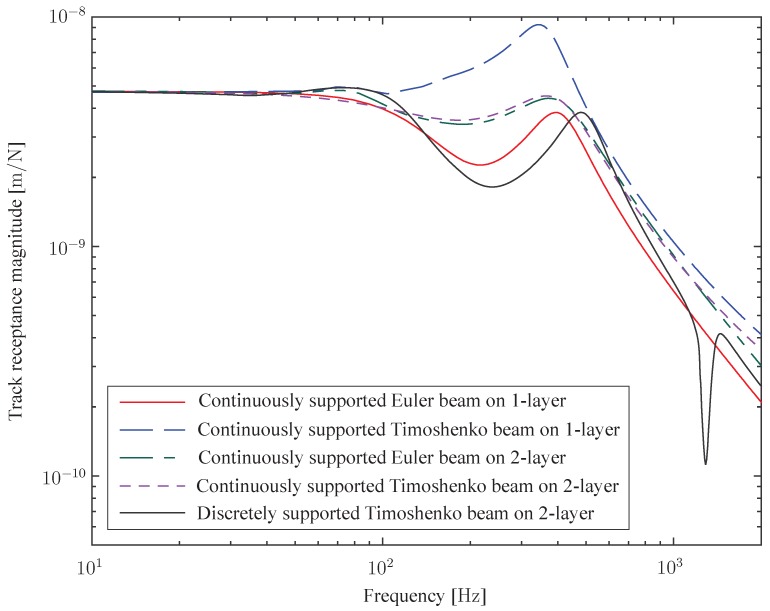
Track receptances calculated using numerical codes.

The simplest model to predict the track deflection is based on an Euler elastic beam resting on a Winkler foundation, which represents a linear stiffness distributed below the rail, taking into account the overall stiffness of the railpads, ballast and foundation (Figure 8a). Although this representation is a drastic simplification, it yields a correct dynamic response of track at low and medium frequencies [27,28]. To overcome this, the next level of refinement consists in modelling the effect of sleeper mass, either using a continuous support (Figure 8b) or a discrete support (Figure 8c). The latter offers the possibility to take into account a different track flexibility above a sleeper or in between two sleepers [29]. Multiple layer models are considered as an improvement over single layer models, as the ballast is modelling in greater detail [26] or the foundation effect on the track response at low frequencies is included [30,31] using either a condensed form for the soil (Figure 8d) or a complete half-space medium (Figure 8e). For some application cases where the stress or strain field are needed in the entire rail, a complete 3D model of the rails is unavoidable or, at the very least, a rail model must be employed providing the stress/strain distribution along the rail height and length. The discrete supports can be modelled by simple equivalent springs in order to reduce the computational cost (time and hardware) imposed by the high number of rail elements. Figure 9 plots typical vertical track receptances for various models. The three aforementioned track modes are seen in this figure: in-phase rail and sleepers mode around 80Hz (highly damped), in-opposite rail and sleepers mode around 400Hz (less damped) and the pinned–pinned mode at 1000Hz; it should be noted that a discretely supported model is the one that provides an accurate description of this last mode (the curve on display is related to a response above a sleeper, explaining the prominent anti-resonance).

Another challenge is to model the dynamic effect of the vehicle, in addition to the dominant frequency excitation imposed by the vehicle geometry configuration. As an alternative to a moving mass load, the multibody approach, which considers some vehicle parts as rigid, offers an elegant solution and the best compromise between accuracy and computational time. It is able to capture the large scale vehicle modes since this simulation allows large displacements and rotations between individual bodies. The main difficulty resides in coupling the vehicle (through the contact elements, the wheels) with the track. Works of Nielsen and Abrahamsson [32], Zhai *et al.* [26] and Oscarsson, Andersson and Dahlberg [29,33] efficiently describe the interaction between the track and the vehicle, in order to establish accurate models, and are still considered an accurate coupling solution for vehicle and track subsystems. These models are suitable for the study of wheel/rail impact forces at local defects, either on the wheel surface (e.g., wheel flat [34]) or on the rail (e.g., rail joint [35] or squat [36]). They also evaluate the resulting dynamic track response under different rolling speeds and under different track configurations, including train–bridge interaction [37,38].

## 4. Typical Sensor Configurations

**Figure 10:**
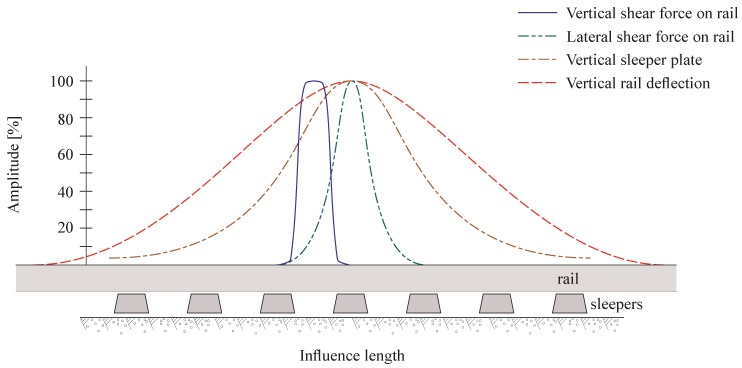
Qualitative influence of load measurement on track (adapted from [40]).

This section is strictly limited to typical and existing solutions dedicated to the wayside track monitoring. Other methods, such as embarked sensors (vehicle-side methods, e.g., [39]), represent also an alternative but are off topic and are not addressed by the present review. Due to the variety of available sensor types, only accelerometers and strain gages are discussed while the next section is devoted to optical-based sensors. A brief description is however given on other sensors. The desired information limits also the use of these transducers: only track dynamics data are evaluated and characterised (vehicle position, speed and acceleration, wheel/rail forces and track deformation). These data are primarily derived from wheel/rail load data provided by the track environment. Figure 10 illustrates the positive effect of these available measurements along the track length.

**Figure 11:**
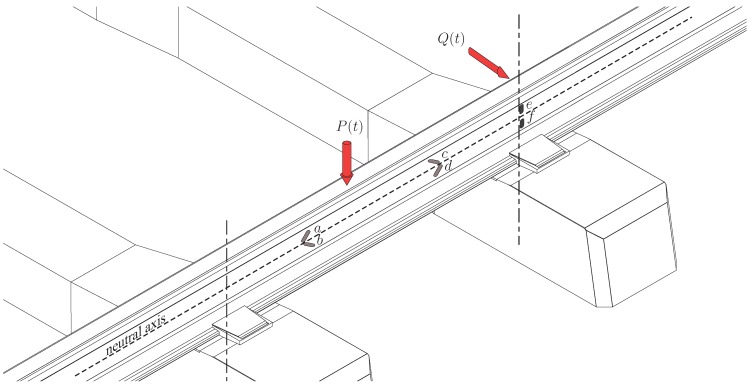
Wheel/rail vertical and lateral forces estimation using strain gage circuits.

Wheel/rail forces are a primary indicator of the vehicle effect. Typically, strain gages are used to estimate this measurand and are placed on the rail (web and foot, the head being sometimes critical for measurement). Knowing the dynamic parameters of the rail (Young’s modulus *E*, Poisson’s ratio *ν*, …) and its geometrical characteristics (web thickness *t*, geometrical moment of inertia *I*, first moment of area *H*, …), the link between stress and strain is easily obtained. This implies that track properties must be defined in sufficient detail to generate accurate parameter values. There are a variety of strain gage circuits that have been used in the context of rail measurements. A complex sensors configuration is shown in Figure 11 where four gages (*a* – *b* – *c* – *d* oriented at 45${}^{{}^{\circ}}$) are placed in the track web on the neutral axis to determine the vertical loading *P*. If possible, the same configuration is used on the other side of the track. If $\epsilon_{i}$ is the strain measured by a sensor (or the sum of the strains measured by this sensor and its adjacent counterpart) and if there is no support (sleeper) under the rail within this gaged region, the vertical wheel/rail force is calculated using

(9) $$\begin{array}{c} P\left(t\right)=\frac{EIt}{n(1+\nu )H}\left(\epsilon_{a}-\epsilon_{b}+\epsilon_{c}-\epsilon_{d}\right) \end{array}$$

where *n* is related to the sensors configuration (on one side n=1, or on both sides of the rail n=2). The placement of these sensors on the neutral axis is the best compromise between sensitivity, cross talk and influence length to have an accurate description of the shear strain $\epsilon_{a}-\epsilon_{b}$ (or $\epsilon_{c}-\epsilon_{d}$). One (two) full bridge set-ups made with the four (eight) sensors easily leads to the desired output between brackets in Equation (9). If there is a sleeper within the test zone, the estimated force is equal to the difference between the wheel/rail force and the sleeper reaction. The proposed configuration provides satisfactory results in many cases. Askarinejad *et al.* [41] used strain gage sensors to detect train dynamic loads and adopted this method to examine the track responses at an insulated rail joint, as well as the response of the continuously welded rail. The gaged area was chosen around the joint in order to measure the wheel loads. Palo *et al.* [42] used the rail force as condition indicators of wheel wear and selected it as a parameter for the condition monitoring of wheel health.

Another configuration is also proposed in Figure 11, based on a lateral shear force circuit (gages *e* and *f*). It utilizes the principle that the shear force in a cantilever beam is proportional to the variation in the bending moment. A calibration is however necessary, using the relationship

(10) $$\begin{array}{c} Q\left(t\right)\propto \epsilon_{f}-\epsilon_{e} \end{array}$$

since the lateral force depends on the whole track configuration. To be suitable and accurate, the gages should be placed over the centre of a sleeper, which constitutes a fixed boundary condition. The first application of this approach was the determination of lateral wheel/rail forces due to the rail flange. The purpose is to prevent for example derailment accidents and abnormal wear (the derailment coefficient Q/P must be kept under the safety limit). Milković *et al.* [43] combined these vertical and lateral configurations in order to estimate the wheel/rail contact forces. Several test cases generated by using finite element analysis were studied using the principle of independent component analysis for successful separation of vertical and lateral contact forces from recorded strain signals. Track buckling can also be monitored by using longitudinal strain gages which allow quantifying rail creep [44]. Dedicated sensors, called MPQY (Multi-Purpose Q and Y load detector) which consists of strain gages directly glued to an intermediary device, can be installed on the rail web and offer the possibility to measure simultaneously the vertical, lateral and longitudinal forces acting on the rail [45]. Conventional methods and the MPQY method for strain measurements on the rail have been compared using finite element analyses [46]. This last work shows the importance of numerical data to calibrate the sensors and to position them properly. Ryjác̆ek and M. Vokác̆ [47] give another example of a combined numerical/experimental assessment, with added value from a sensitivity analysis of several track parameters (notably the ballast stiffness).

**Figure 12:**
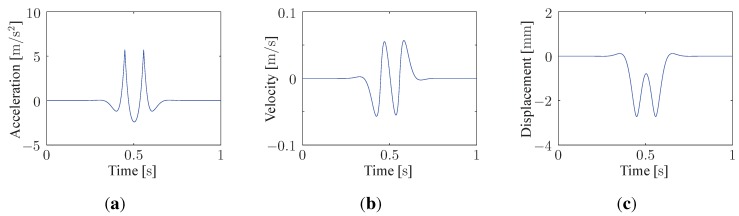
Track deflection due to the passing of a single bogie. (**a**) Acceleration; (**b**) Velocity; (**c**) Displacement.

A variety of motion transducers are suitable to make track deflection measurements. Although displacement transducers yield only to relative displacement measurements, accelerometers represent nowadays the most used absolute motion transducer type due to their easy mounting and their large dynamic and frequency ranges. The use of geophones as an alternative seismic sensor presents some merits: measurement of large displacement amplitudes, no power supply, low cost device, … at the expense of a limited bandwidth (cut-off low-frequency relatively high, typically at 4–12Hz and limited high-frequency, up to maximum 1kHz) and phase errors. Since the cut-off low-frequency is proportional to the inverse root of the seismic mass, geophones with low corner frequencies can be obtained electronically (using an electronic and powered equipment) or numerically (using an appropriate signal filtering, e.g., [48]) correcting the low frequency sensitivity, at the price of higher noise and cost. The corresponding track deflection is then obtained through successive integrations (2 for an accelerometer, 1 for a geophone). This is illustrated in Figure 12. However, robust signal processing techniques are required to avoid non-physical signals associated with the integration constant inherent to the original noisy signal and to the sampling rate. Typical solutions include algorithms that extract the noise considered as a Gaussian distribution [49], methods based on a frequency domain criterion applied on a velocity reconstruction [50] or removing the low-frequency content using adequate digital filtering [51,52]. Alternatively, laser Doppler velocimetry and high-speed video camera can be used to monitor the rail surface motion and to evaluate the rail defection [4,53] but they are dependent on the environmental condition and they cost is relatively high, compared to seismic sensors.

The maximum vertical rail deflection $w_{max}$ can be determined using the theory of a beam continuously supported by a Winkler foundation [54]

(11) $$\begin{array}{c} w_{max}=\frac{P}{8EI\beta^{3}} \end{array}$$

where $\beta =\sqrt[{4}]{\frac{K_{f}}{4EI}}$ is introduced, representing the ratio of flexibility between the foundation and the rail. $K_{f}$ is the foundation stiffness per unit of length. This formula does not take into account the influence of adjacent wheel (e.g., in a bogie, as illustrated in Figure 12). Equation (11) is also an approximation of the whole vertical track displacement.

Several relative and absolute displacements are of interest in the characterisation of the dynamic response of a track: the rail vertical absolute deflection, the rail head and foot lateral deflection, the sleeper lateral and vertical deflections, the rail rotation (difference of rail head and foot lateral deflections) and the dynamic gage (difference of left and right head displacements). When possible, direct displacement measurements are preferable, especially for long-term installations, but they are subject to some drawbacks:

- Contacting displacement transducers like LVDT yield a relative motion because of how they are mounted. They have a limited frequency bandwidth (modern LVDTs have a usable bandwidth from 0 to 1kHz [49]) and may be not suitable for some applications where the frequencies of interest are outside this range. They also need to be calibrated during installation.
- Non-contacting transducers like capacitance-type transducers present excellent stability and repeatability and require little maintenance. However, a particular attention should be paid to their calibration to assure proper linearity and sensitivity. They are also sensitive to electrical noise and to temperature.
- Most often, a specific and dedicated mounting arrangement is necessary, which complicates installation.

The use of accelerometers, combined with efficient signal processing, offer an elegant alternative to continuous displacement measurement. In addition, accelerometers can detect impending adverse changes in a structure under service conditions. In association with anti-aliasing filters, they provide an accurate assessment of frequency content and vibration levels. Kouroussis *et al.* [55] showed that a continuous measurement of train-generated vibrations using accelerometers on the track and even on the ground provides a semi-remote and non-invasive monitoring of train velocities. The proposed speed calculation method is based on the vibration spectrum and on the knowledge of the main dimensions of trains (knowing the modulation effects presented in Section 2). Table 1 summarizes the metrological performances of typical transducers.

Where possible, a mix of strain gages and accelerometers can be considered for the conditioning and monitoring of vibration signals, as proposed in [41,56]. Modern accelerometers such as small micro electro-mechanical systems (MEMS) represent interesting and economically viable transducers which can be bundled together with other sensors (inclinometers, strain gages, distance sensors, …) into compact systems that can cost-effectively monitor railway infrastructures using wireless networks [57,58].

**Table 1:** Performances of typical transducers for railway monitoring.

	Contributing Parameters	Requirements/Limitations
**Strain gage**	Wheel/rail force	Accurate positioning (neutral axis)
	Vehicle position	Sensitive to electromagnetic interferences
		Difficulty of gluing and/or soldering
		(except waterproof or embedded sensor systems)
**Accelerometers**	Track deflection	Absolute motion
	Vehicle position	Sensitive to external excitation
		Specific signal processing
**Geophones**	Track deflection	Absolute motion
	Vehicle position	Sensitive to external excitation
		Limited frequency range
**LVDT**	Track deformation	Specific mounting
		Limited frequency range
**MDD**	Foundation deflections	Specific installation

## 5. Using Optical Fibre-Based Sensors as an Alternative

Besides electrical detection systems, numerous research groups have demonstrated the usefulness of the optical fibre technology to monitor railway infrastructures. With their use, one can determine the structural health of the tracks and its evolution with time, while measuring the traffic. And as such, the maintenance operations can be improved, increasing both the safety level and the availability.

Optical fibres are mainly used for telecommunication purpose. They are made of two concentric cylindrical layers, so-called core and cladding, and guide light thanks to a slight refractive index difference between these two layers. Most often, the cladding is in pure silica while the core is made of silica doped with germanium oxide. There are different kinds of optical fibres but the most often used are single-mode fibres, composed of a 8µm core surrounded by a 125µm cladding. An additional 250µm polymer layer confers to the whole optical fibre an axial strain resistance superior to the one of a steel wire of the same cross-section. Historically, the idea to use optical fibres as sensor elements arose from the wish to isolate optical fibre communications from unwanted fluctuations caused by external perturbations such as temperature changes. Since then, optical fibre sensors have emerged in numerous applications. Whatever the transduction mechanism, they bring all the advantages inherent to the use of optical fibres, such as remote operation over long distances (several tens of kilometres), light weight and ease of installation, good resistance to corrosion and high temperatures, immunity to electromagnetic interferences, …

An overview of the literature shows that the most straightforward use of optical fibres for railway applications remains the broken rail detection where lengths of optical fibres are glued along the tracks and act as a fuse [59]. Using a time domain reflectometric technique as a demodulation process, the rail break is detected by the absence of reflection due to the optical fibre failure. More interestingly, strain sensors can be realized from optical fibres, allowing them to be used for railway traffic monitoring. Among the possible configurations, Bragg grating-based and Brillouin-based sensors were the most developed in this field and will thus retain our attention in the following.

Fibre Bragg gratings (FBGs) are photo-inscribed by a lateral illumination of the optical fibre with an interference pattern of ultraviolet light (≈240nm wavelength). They correspond to a permanent and periodic refractive index modulation of the fibre core along the propagation axis. They are wavelength-selective mirrors, reflecting a narrowband resonance centred on the so-called Bragg wavelength. The latter is given by

(12) $$\begin{array}{c} \lambda_{\mathrm{Bragg}}=2n_{\mathrm{eff}}\Lambda \end{array}$$

where $n_{\mathrm{eff}}$ is the effective refractive index of the fibre core and Λ is the grating period. Any change in the ambient temperature or strain applied on the FBG modifies these physical parameters, which in turn induces a wavelength shift (≈$10\;\mathrm{pm}{/}^{{}^{\circ}}C$ or ≈1 pm/µϵ). In the elastic region, the Bragg wavelength shift is linear and without hysteresis, yielding an easy-to-process response. The read-out technique is based on the monitoring of the Bragg wavelength shift in the FBG amplitude spectrum, which can be performed using different approaches, as described in [60,61,62]. Contrary to conventional strain gages, FBGs allow an easy wavelength multiplexing with a single optical fibre connecting several tens of FBGs photo-written at different Bragg wavelengths to prevent spectral shadowing. Sharing a single measurement device for several tens of sensing points yields a competitive cost per sensor channel. FBGs also provide a resolution of the order of 1µϵ when used for axial strain sensing with commercial dedicated interrogators. Finally, depending on the interrogator operating principle, they can be interrogated at a very high speed, typically up to several kHz.

**Figure 13:**
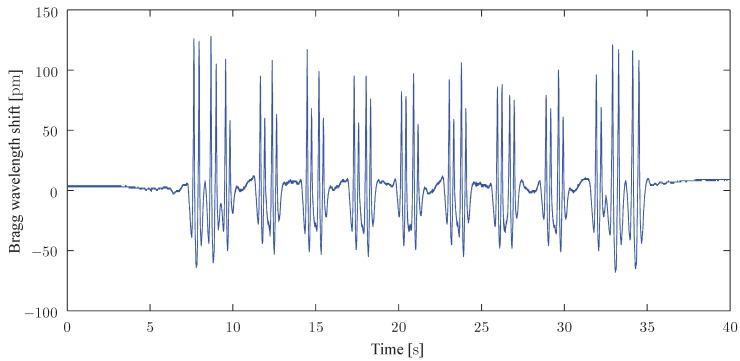
Trace (Bragg wavelength shift with time) of an FBG glued on the foot of a 50E2 rail and subject to a train circulating at 40km/h.

Many works using FBGs sensors for railway applications have been reported so far [63,64,65]. The main contributions come from the Polytechnic University of Hong-Kong (Tam *et al.*), the Universita Degli Studi del Sannio in Benevento, Italy (Cusano *et al.*) and the Universidad de Alcala, Alcala de Henares, Spain (Gonzalez-Herraez *et al.*). In particular, it was shown that a single FBG glued to the rail can provide useful information about the occupation state, the train composition (through axle counting and weighing in motion), its velocity and acceleration [63]. The group of M. Gonzalez-Herraez demonstrated that, depending on the parameters to be measured, particular orientations and locations of sensors should be considered depending on the measurement function. In doing so, they have achieved the detection of flat wheels [66]. The team of A. Cusano proposed an optimized packaging allowing the fastening of the sensor to the foot of the rail. Compared to gluing, this adapted packaging offers rapid and non-invasive installation, preventing the need to drill the rail [65]. Sensors are also easily removable, which is an undeniable asset. We have started to work on such FBG sensor developments since one year now. Figure 13 shows the evolution with time of the wavelength shift of a 4mm long bare FBG glued with UV curing urethane acrylate on the foot of a 50E2 type rail (close to classical UIC 50 rail section and commonly used in Belgium). This type of glue was privileged in our work as it offers the best compatibility between glass and metal while shielding the grating from moisture. The passage of a train circulating at 40km/h measured with an FBGScan interrogator sampled at 1kHz can be observed in Figure 13. The obtained shape can be directly compared to the track deflection plotted in Figure 12c. FBGs can be wavelength-multiplexed in a single optical fibre. Currently available competitive interrogators in terms of performances and cost have operational bandwidths (80–100nm) suitable to interrogate up to 50 sensors cascaded along an optical fibre cable. Obviously, the quasi-distributed nature depends on the spacing between adjacent gratings that can vary from several centimetres to several hundreds of meters.

Distributed sensors relying on the Brillouin effect are based on the inelastic interaction between light travelling in the fibre core and the acoustic phonons. It yields a well-defined resonance that shifts with temperature ($1\;\mathrm{MHz}{/}^{{}^{\circ}}C$) and axial strain (1MHz/20µϵ). Two techniques are available for demodulation, namely the Brillouin Optical Time Domain reflectometry (BOTDR) and the Brillouin Optical Time Domain Analysis (BOTDA). The principal asset of the BOTDR is that it only requires the access to one fibre end. However, this comes with a lower efficiency, which leads to long measurement times. The BOTDA, where the fibre is looped to the instrument, offers a much better signal-to-noise ratio. Due to Brillouin amplification, the BOTDA provides useful signals with intensities of two orders of magnitude higher than the inherent Rayleigh backscattering level resulting from inhomogeneities present in the fibre core. Standard commercial systems provide measurements in a 30–50km range with a 1m resolution (or even better, but then for a smaller range). The resolution achieved on the measurement of the Brillouin frequency remains about 1MHz, which is equivalent to an uncertainty of 1 ${}^{{}^{\circ}}C$ in temperature and 20µϵ in strain.

There are a few achievements about the use of distributed sensors for railway monitoring applications. In [67], the results of a dynamic distributed strain measurement over a 70m monitored section of rail track were reported. Train passages were revealed by the system but without identification of the train characteristics. In [68], high spatial resolution strain measurements over a 2.8m rail track length were reported. The sensor was able to retrieve the strain distribution induced by an imposed load. However, only quasi-static tests were reported and there was no attempt to demonstrate the train identification capability of the system. Using a BOTDA, Minardo *et al.* [69] recently demonstrated the real-time monitoring of railway traffic with a 31Hz acquisition rate and a 1m spatial resolution. The data acquired by the sensor have demonstrated its capability of retrieving useful information, such as train identification, axle counting, speed detection and dynamic load estimation. Besides their use directly on tracks, optical fibre sensors were also reported to monitor railway infrastructures, such as bridges and viaducts [70], and for current measurement in the catenary [71].

Besides these techniques, Rayleigh scattering based distributed acoustic sensing (DAS) can also be implemented. In this case, a coherent laser pulse is launched into an optic fibre and scattering within the fibre causes the fibre to act as a distributed interferometer with a gauge length close to the pulse length [72]. The reflected light intensity is recorded as a function of time. A new pulse is launched when the former one has made a complete round-trip in the fibre under test. Changes in the reflected intensity of successive pulses from a given fibre section are caused by changes in the optical path length of that section. This system is very sensitive to both strain and temperature variations and measurements can be made simultaneously at all sections of the fibre. With a suitable analysis software, continuous monitoring is possible. The companies FOTECH Solutions and Optasense have recently demonstrated that the optical communication fibres buried close to the railway tracks can be used as a DAS for railway traffic monitoring [73,74].

Table 2 summarizes the main performances that could be expected from optical fibre sensor technologies when used as trackside monitoring solutions.

**Table 2:** Performances of optical fibre transducers for railway monitoring.

	Transduction Mechanism	Sensor Location	Sensor Fixation	Spatial Resolution
**Bragg**	Axial strain induces a Bragg wavelength shift	On the rail or on the sleeper	With glue, screws, welding or with dedicated magnetic or mechanical patch	Quasi-distributed sensing (maximum 50 gratings per channel
**Brillouin**	Axial strain induces a shift of the Brillouin frequency	On the rail	With glue	Distributed sensing (≈1m)
**Acoustic**	Acoustic pressure induces a change of the Rayleigh backscatter intensity	On the rail, close to the rail or even buried	With glue if on the rail	Distributed sensing (≈1m)

## 6. Discussion and Useful Guidance

**Figure 14:**
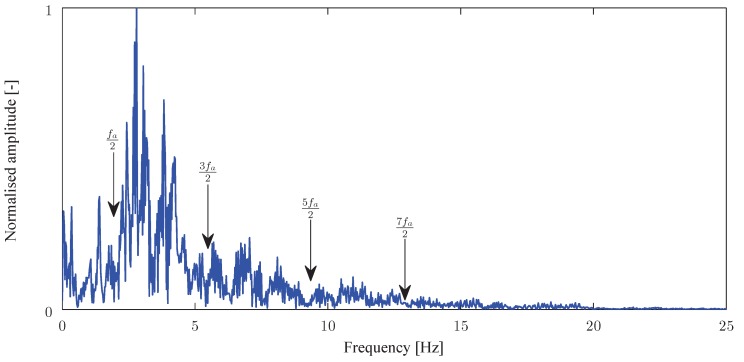
Frequency content of the signal presented in Figure 13 ($L_{a}=3\;m$).

The analysis of track monitoring sensors revealed that several solutions exist, depending on the transduction mechanism and the expected track information. Additional points of interest include:

- The utility of a prediction model has been pointed out in order to assess the optimal positioning and orientation of sensors. Increasing the complexity of a numerical model goes hand in hand with the need for more track parameters (e.g., railpad, sleeper or foundation behaviour) and an increase of computational burden, which often limit the analysis to simple cases.
- The knowledge of track dynamics allows the understanding of some specific phenomena. For example, the spectral analysis of the recorded time history presented in Figure 13 is plotted in Figure 14. The expected information is ranged between 0 and 25Hz due to the low vehicle speed (40km/h). As described in Section 2, an amplitude modulation is clearly visible and some information can be deduced from these periodicities (for example, at frequencies $\frac{2k+1}{2}f_{a}$, the amplitude tends towards zero).
- Some important requirements for classical sensors (strain gauges) can be used as a guidance for the positioning of fibre-based sensors and the analysis of resulting signals. Position and orientation can be borrowed from the experience of strain gauges and applied to fibre sensor measuring the local deformation.
- The mounting of the sensors is also of great importance. Although cementing and screwing are the most commonly adopted solution, other mountings (welding, magnetic attaching, clamping, …) are also of interest. It appears that there is no universal solution for sensor packaging but some precautions and requirements must be taken: a correct strain transfer (rail polishing in the case of gluing), an ease of installation, a minimum of robustness towards weather and resistance to rail maintenance, a replacement without damaging the sensor, …
- A high reliability is required in railway industry, according to IEC 61508 [75] and IEC 62279 [76] standards (IEC 62279 provides a specific interpretation of IEC 61508 for railway applications). A risk assessment effort yields a target safety integrity level (SIL) with an expected probability of failure per hour less than $10^{-9}$ (SIL4, which is the most dependable, has become a requirement in railway to attain in regards to a system’s development).

## 7. Conclusions

This paper has presented an overview about the static and dynamic behaviour of ballasted railway tracks, allowing the positioning of the problem and the definition of sensor requirements. It has then shown how to estimate stress transfer from the train passage to the track using predictive numerical models. From classical strain gauges to their optical fibre counterparts allowing miniaturized sensors to be cascaded over long distances in a single wire, a literature overview has finally been made about available solutions to monitor train traffic. This short description will be of benefit to the railway community as a whole, as a solid working base for further advanced sensor developments. With the expansion of high speed trains and the challenges that remain to tackle, such as broken rail detection independent of the signalling system, it is likely that this review article will foster further research and developments in this continuously growing field.
